# An international wheat diversity panel reveals novel sources of genetic resistance to tan spot in Australia

**DOI:** 10.1007/s00122-023-04332-y

**Published:** 2023-03-13

**Authors:** Julian Taylor, Dorthe Jorgensen, Caroline S. Moffat, Ken J. Chalmers, Rebecca Fox, Grant J. Hollaway, Melissa J. Cook, Stephen M. Neate, Pao Theen See, Manisha Shankar

**Affiliations:** 1 School of Agriculture, Food and Wine, Waite Research Institute, University of Adelaide, Glen Osmond, SA 5064 Australia; 2 Department of Primary Industries and Regional Development, Agriculture and Food, 3 Baron Hay Ct, South Perth, WA 6151 Australia; 3 Centre for Crop Disease and Management, School of Molecular and Life Sciences, Curtin University, Kent St, Bentley, WA 6102 Australia; 4 Agriculture Victoria, Private Bag 260, Horsham, VIC 3401 Australia; 5 Centre for Crop Health, University of Southern Queensland, Toowoomba, QLD 4350 Australia; 6 School of Agriculture and Environment, University of Western Australia, 35 Stirling Hwy, Crawley, WA 6009 Australia

## Abstract

**Key message:**

Novel sources of genetic resistance to tan spot in Australia have been discovered using one-step GWAS and genomic prediction models that accounts for additive and non-additive genetic variation.

**Abstract:**

Tan spot is a foliar disease in wheat caused by the fungal pathogen *Pyrenophora tritici-repentis* (Ptr) and has been reported to generate up to 50% yield losses under favourable disease conditions. Although farming management practices are available to reduce disease, the most economically sustainable approach is establishing genetic resistance through plant breeding. To further understand the genetic basis for disease resistance, we conducted a phenotypic and genetic analysis study using an international diversity panel of 192 wheat lines from the Maize and Wheat Improvement Centre (CIMMYT), the International Centre for Agriculture in the Dry Areas (ICARDA) and Australian (AUS) wheat research programmes. The panel was evaluated using Australian Ptr isolates in 12 experiments conducted in three Australian locations over two years, with assessment for tan spot symptoms at various plant development stages. Phenotypic modelling indicated high heritability for nearly all tan spot traits with ICARDA lines displaying the greatest average resistance. We then conducted a one-step whole-genome analysis of each trait using a high-density SNP array, revealing a large number of highly significant QTL exhibiting a distinct lack of repeatability across the traits. To better summarise the genetic resistance of the lines, a one-step genomic prediction of each tan spot trait was conducted by combining the additive and non-additive predicted genetic effects of the lines. This revealed multiple CIMMYT lines with broad genetic resistance across the developmental stages of the plant which can be utilised in Australian wheat breeding programmes to improve tan spot disease resistance.

**Supplementary Information:**

The online version contains supplementary material available at 10.1007/s00122-023-04332-y.

## Introduction

Tan spot or ‘yellow leaf spot’ is a foliar disease of wheat (*Triticum aestivum* L.) caused by the necrotrophic fungal pathogen *Pyrenophora tritici-repentis* (Ptr) of the order Pleosporales. It may cause substantial yield losses by decreasing both kernel weight and numbers of grain per head (Shabeer and Bockus 1988). In Australia, the reduction in annual gross wheat yield attributed to losses by tan spot has been estimated as high as 3% (Murray and Brennan 2009) with more recent research suggesting losses may be as high as 4.5–6% in North and South American wheat growing regions (Savary et al. 2019). These gross yield reductions translate to over a billion dollars in annual revenue losses globally.

Within local cropping systems, yield losses can be intensified by a combination of farm management practices such as stubble retention or minimum tillage and the cultivation of tan spot susceptible wheat varieties (Rees and Platz 1983; Carignano et al. 2008). With infected wheat residues present in a newly sown field, germinating seedlings can be exposed to early disease pressure. Upon occurrence of significant rainfall events the disease can progress rapidly and significant yield losses of up to 50% are possible (Rees and Platz 1983; Bhathal et al. 2003; Carignano et al. 2008). Thus, the development and use of tan spot resistant varieties to circumvent these ongoing issues offers a long-term approach.

The tan spot host–pathogen interaction is complex. The pathogen is known to secrete three effectors (host-selective toxins), Ptr ToxA, Ptr ToxB and Ptr ToxC, that interact with host specific sensitivity genes to cause necrosis or chlorosis (Lamari and Bernier 1989; Lamari et al. 2005; Kamel et. al 2019). Triggering of plant susceptibility through the interaction of the host with any combination of these effectors is known as the inverse gene-for-gene model (Fenton et al. 2009) due to the pathogen recognition of host signals. Numerous host sensitivity loci have been previously identified and directly affect effector sensitivity (Faris 1996; Effertz et al. 2002; Friesen and Faris 2004; Tadesse et al. 2006a, 2006b; Singh et al. 2009). Wheat lines harbouring the major host sensitivity gene *Tsn1* on chromosome 5BL are sensitive to Ptr ToxA and exhibit strong necrosis following infection with ToxA-producing Ptr isolates (Faris 1996), while the chlorotic effects induced by Ptr ToxB and Ptr ToxC are observed in wheat lines possessing the *Tsc2* and *Tsc1* loci on chromosomes 2B and 1A, respectively (Effertz et al. 2002; Friesen and Faris 2004; Corsi et al. 2020). Other useful non-race specific quantitative trait loci (QTL) have also been reported and may, in combination with known genes, be useful for breeding more durably resistant varieties (Shankar et al. 2017; Dinglasan et al. 2018; Liu et al. 2020; Phuke et al. 2020).

In the exploration for novel sources of genetic resistance to tan spot, historical wheat diversity panels have been phenotypically screened using various pathogen isolates and Ptr effector bioassays (Abdullah et al. 2017; Dinglasan et al. 2018). Abdullah et al. (2017) conducted research using the Ptr race 1 isolate from USA and, as expected, the majority of wheat cultivars or genotypes developed before the 1950s green revolution showed susceptibility. Dinglasan et al. (2018) focussed on evaluating the Vavilov collection using a mixture of Australian Ptr isolates where the race of the individual isolates was not determined but the presence of ToxA gene and the absence of ToxB was confirmed in these isolates. Although they found diverse reactions across landraces in the panel, including enhanced level of resistance observed for all growth stages, they are not well suited for commercial breeding due to the lack of adaptability to high input agriculture. For those studies in which genotyping information was available, genome wide association studies (GWAS) have been conducted to determine significant genomic regions linked to tan spot resistance (Gurung et al. 2014; Kollers et al. 2014; Dinglasan et al. 2018; Juliana et al. 2018; Phuke et al. 2020; Lozano-Ramírez et al. 2022). Gurung et al. (2014), Juliana et al. (2018), Phuke et al. (2020) and Lozano-Ramírez et al. (2022) reported small numbers of moderately significant loci based on disease assessment of tan spot on seedlings grown in controlled environments, whereas Dinglasan et al. (2018) conducted GWAS of tan spot traits collected at seedling and adult growth stages in multiple glasshouse and field experiments. Although some novel QTL have been identified, most of the studies have demonstrated the highly polygenic nature of tan spot disease in wheat across varying environments.

For wheat traits that are known to be highly polygenic, genomic prediction has been proven to provide a more complete measure of the genetic performance of the population (Norman et al. 2017; Tsai et al. 2020). In the context of crop disease, Poland and Rutkoski (2016) recognised the potential of genomic prediction for breeding quantitative disease resistance in tandem with other industry relevant traits such as yield. In tan spot disease research, studies are now starting to emerge that focus on genomic prediction of disease severity to understand genetic resistance across varying environments (Poland and Rutkoski 2016; Juliana et al. 2017; Muqaddasi et al. 2021). However, these initial studies have focussed on determining an appropriate prediction modelling approach that provides the greatest accuracy for genomic selection purposes rather than interpreting the overall prediction results for useful line selection.

In this study, we assembled an international wheat diversity (IWD) panel with varying levels of tan spot resistance consisting of bread wheat lines from the International Maize and Wheat Improvement Centre (CIMMYT), the International Centre for Agriculture in the Dry Areas (ICARDA) and Australia (AUS). These lines were then screened for tan spot severity using Australian Ptr isolates in various controlled environment and field locations around Australia, with disease assessment conducted at various stages of plant development. Experiments were repeated over two consecutive years. In addition, the IWD panel was screened for sensitivity to Ptr ToxA and Ptr ToxB using purified effector proteins in plant bioassays and assessed for necrosis and chlorosis. Unfortunately, screening for Ptr ToxC sensitivity using plant bioassay was not possible in this study as the structure of Ptr ToxC has yet to be elucidated. The IWD panel was then genotyped using the 90 K Illumina iSelect SNP array designed for wheat (Wang et al. 2014). For each of the tan spot traits, a GWAS was conducted using an efficient one-step analysis approach to ensure all genetic and non-genetic sources of variation were accounted for simultaneously. This allowed for the rapid identification of significant loci associated with multiple tan spot traits and also identified many singleton QTL. To further understand whether individual lines exhibited broad resistance to tan spot in Australia, we also conducted a genomic prediction of the tan spot traits. From this analysis, we focussed on providing a useful summary of the IWD panel line predictions to allow easy selection of lines for future use in Australian wheat breeding programmes.

## Material and methods

### Plant material

The IWD panel of 192 lines used in this research was chosen from a larger set of 1000 lines screened for tan spot resistance against a mixture of contemporary local Ptr isolates from 2010 to 2014 at various growth stages and environments at South Perth. The IWD panel was specifically selected to contain several region specific sub-populations where the lines within each region represented a range of resistance levels and pedigree diversity. The sub-populations of the panel consisted of: 47 Australian (AUS) lines (AUS-1 to AUS-47), including 23 Australian commercial wheat varieties with varying levels of tan spot resistance, 121 lines from the CIMMYT bread wheat breeding programme (CI-1 to CI-121) and 24 lines from the ICARDA bread wheat breeding programme (IC-1 to IC-24). Full names of each line from each sub-population region are given in Supplementary Table S1.

The IWD panel was phenotyped for resistance against a mixture of local isolates at various growth stages and environments in 2015 and 2016 at South Perth, Western Australia (S31°59.20’, E115°53.13’); at Horsham, Victoria (S36°44.61’, E142°6.68’); and at Toowoomba, Queensland (S27° 32.00’, E151° 56.15’). Additionally, the IWD panel was screened for sensitivity to Ptr ToxA and Ptr ToxB at South Perth, Western Australia (S31°59.20’, E115°53.13’).

### Experimental designs

For all controlled environment, glasshouse and field trials conducted in this research, experimental designs were generated as spatially optimal row-column designs with layout configurations defined in Table 1. In each experiment, the experimental units within a Block were allocated a combination of the complete set of IWD panel lines with additional spread of local control or filler varieties to generate a rectangular grid arrangement.

**Table 1:** Summary of the spatially optimal row-column designs conducted at each experimental location in each year

Exp. type	Location	Year	Environ. type	Inoc.type	#R	#B	#CB	#BR	#BC	#EU
Tan Spot Sev	Horsham, VIC	2015	Glasshouse	Isolate	4	4	11	7	29	812
			Field	Isolate	3	3	28	10	22	660
		2016	Glasshouse	Isolate	3	3	32	14	16	672
			Field	Isolate	3	3	28	10	22	660
	South Perth, WA	2015	Cont. Env	Isolate	3	3	4	7	28	588
			Field	Isolate	3	3	8	10	20	600
		2016	Cont. Env	Isolate	3	3	4	7	28	588
			Field	Isolate	3	3	8	10	20	600
	Toowoomba, QLD	2015	Cont. Env	Isolate	2	2	16	18	12	432
			Field	Isolate	3	3	–	12	16	576
		2016	Cont. Env	Isolate	2	2	16	18	12	432
			Field	Isolate	2.5	3	16	11	16	528
Bioassay	South Perth, WA	2019	Contr. Env	ToxA Effector	3	3	24	6	36	648
				ToxB Effector	3	3	24	6	36	648

#R represents the number of replicates of the IWD panel (AUS, CI, IC); the number of blocks (#B); the number of controls and fillers within each block (#CB); the number of rows in each Block (#BR); the number of columns in each Block (#BC); the number of total experimental units (#EU)

All experimental designs were computationally generated using the model-based optimal design R package odw (Butler 2021). To achieve this an initial design data frame was generated that contained Row, Range and Block factors with a Variety factor that includes each replicate set of lines exclusively placed in individual design blocks. All of the factors of the design frame were then specified as an additive set of random model terms in the main function call of the package. The call also specifies that permuting of the rows of the initial design data frame is restricted to unique genotype swaps within block levels specified in the Block factor. We used the inbuilt Tabu search algorithm for conducting swaps within a localised neighbourhood and assessment of objective function improvement. An important feature of the design specification was the inclusion of spatial optimality or minimisation of line allocations across the row and ranges of the experiment to provide protection against reduction in accuracy of line effects if environmental trends were present across the experiment. This was achieved by fixing the numerical variances of the Row and Range terms to a substantially large number to force the optimisation algorithm to favour designs where allocation of the replicates of the same variety were evenly ameliorated in both directions of the experimental layout.

### Tan spot phenotyping trials

A complete description of the protocols relating to the phenotyping experiments conducted in 2015 and 2016 at each location is given in the proceeding sections, and the collated summary of these protocols, as well as information about numerical and analytical aspects of the resulting tan spot severity traits, is given in Table 2. The final column in Table 2 indicates each resulting tan spot severity trait has been given a unique alphanumeric code and these codes are used in the proceeding text, graphics and other tables to provide consistency throughout the article.

**Table 2:** Across the three experimental locations in 2015 and 2016, a summary of experimental protocols undertaken to generate each tan spot symptom severity trait as well as numerical and analytical information about each of the resulting traits

Location	Year	Environment type	Stage(s) of inoculation	Growth stage at assessment	Time of assessment	Plant part assessed	Assessment measure	${H}^{2}$	Transform	Code
Horsham, VIC	2015	Glasshouse	Seedling	Seedling	8 days after inoculation	Inoculated leaves	Rating (1–9)	0.885	Original	*A*
		Field	Seedling	Adult	Stem elongation	Middle leaf layers	Rating (1–9)	0.717	Original	*B*
			Seedling	Adult	Booting	Middle leaf layers	Rating (1–9)	0.650	Original	*C*
	2016	Glasshouse	Seedling	Seedling	8 days after inoculation	Whole plant	Rating (1–9)	0.123	Original	*D*
		Field	Seedling	Adult	Early stem elongation	Middle leaf layers	Rating (1–9)	0.778	Original	*E*
			Seedling	Adult	Early booting	Middle leaf layers	Rating (1–9)	0.762	Original	*F*
			Seedling	Adult	Late booting	Middle leaf layers	Rating (1–9)	0.791	Original	*G*
			Seedling	Adult	Early anthesis	Middle leaf layers	Rating (1–9)	0.752	Original	*H*
			Seedling	Adult	Late anthesis	Top 4 leaf layers	Rating (1–9)	0.758	Original	*I*
South Perth, WA	2015	Controlled	Seedling	Seedling	9 days after inoculation	Inoculated leaves	Rating (0–5)	0.859	Original	*J*
			Seedling; heading	Adult	14 days after second inoculation	Flag leaves	Percentage	0.717	Logit	*K*
		Field	Heading	Adult	390 °C thermal d after inoculation	Flag leaves	Percentage	0.899	Logit	*L*
	2016	Controlled	Seedling	Seedling	9 days after inoculation	Inoculated leaves	Rating (0–5)	0.835	Original	*M*
			Seedling; heading	Adult	14 days after second inoculation	Flag leaves	Percentage	0.766	Logit	*N*
		Field	Heading	Adult	390 °C thermal d after inoculation	Flag leaves	Percentage	0.854	Logit	*O*
Toowoomba, QLD	2015	Controlled	Seedling	Seedling	9 days after inoculation	Inoculated leaves	Rating (1–9)	0.780	Original	*P*
			Seedling; heading	Adult	14 days after second inoculation	Top 2 leaf layers	Rating (1–9)	0.547	Original	*Q*
		Field	Seedling	Seedling	Early tillering	Whole plants	Rating (1–9)	0.811	Original	*R*
			Seedling	Adult	Anthesis	Top 2 leaf layers	Rating (1–9)	0.759	Original	*S*
	2016	Controlled	Seedling	Seedling	9 days after inoculation	Inoculated leaves	Rating (1–9)	0.395	Original	*T*
			Seedling; heading	Adult	14 days after second inoculation	Top 2 leaf layers	Rating (1–9)	0.462	Original	*U*
		Field	Seedling	Seedling	Early tillering	Whole plants	Rating (1–9)	0.557	Original	*V*
			Seedling	Adult	Anthesis	Top 2 leaf layers	Rating (1–9)	0.376	Original	*W*

${H}^{2}$ refers to a generalised broad sense heritability calculated using Cullis et al. (2006). The final column represents the alphanumeric code used for unique identification of each tan spot symptom severity trait, and these are used in the text, various graphics and tables contained within this article

#### South Perth, Western Australia

The IWD panel was assessed at the seedling and adult plant stages under controlled environment conditions and at the adult plant stage in an irrigated field nursery in both years. For all trials, an equal mix of the following ten contemporary isolates obtained from the Western Australian Plant Pathology Reference Culture Collection (WAC) was used for inoculation: WAC13611, WAC13614, WAC13769, WAC13651, WAC13767, WAC13768, WAC13770, WAC13772, WAC13774 and WAC13793. Inoculum was prepared as described by Shankar et al. (2017). The conidial suspension concentration was adjusted to 3000 spores/ml in 0.5% gelatine solution for all inoculations.

For the controlled environment experiments in both years, experimental design blocks were arranged on separate benches. Lines were grown in a controlled environment with 24/22 °C day/night temperatures and 12 h of natural day light. Four seeds per line were sown within each 120 mm diameter pot containing a sand-loam mix with 1 g of Osmocote (slow-release fertiliser). At Zadoks growth stage 12.5 (Zadoks et al. 1974) seedlings were spray-inoculated to run-off with the conidial suspension as described above. Inoculated plants were incubated for 24 h in a humidifier with 95–100% relative humidity while maintaining the same block structure and row by column layout. Nine days after inoculation, leaves that had been fully emerged at inoculation were rated for severity on a 0–5 scale in 0.5 increments, where 0 is no disease and 5 is high disease. The severity scale uses a combination of lesion type (Lamari and Bernier 1989), lesion size, and percentage leaf area diseased relative to the response of susceptible controls. Immediately after the initial disease rating, plants were provided with a 20 h photoperiod consisting of 12 h of natural day light and 8 h of high-pressure sodium light with an active radiation of 400–500 μmol m^−2^ s^−1^. Plants were fertilised with soluble all-purpose Thrive N/P/K 25:5:8.8 (Yates Australia, Padstow NSW) at a concentration of 0.8 g/L and a rate of 60 mL/pot on a weekly basis and with a trace element solution of Liberal BMX (BASF) at a concentration of 0.5 g/L and a rate of 30 ml/pot on a fortnightly basis. At heading (Zadoks 55), flag leaves of individual plants in each pot were inoculated as described above. Fourteen days after this inoculation, percentage leaf area diseased (PLAD) was visually scored on the flag leaves and PLAD values were averaged over the sampled leaves for each pot to ensure a single numerical tan spot symptom severity value was attributed to each experimental unit.

Field experiments were conducted using methods that mitigated possible confounding effects of plant maturity and height on disease expression at the adult plant stage as described by Shankar et al. (2017). Plots consisted of two 10 cm rows 10 cm apart, with up to 10 seeds sown per row and with 30 cm between adjacent plots. Plots were fertilised with a mixture of superphosphate, urea and potash (6:4:1) at a rate of 100 kg/ha at planting and at 8 weeks after sowing. Plots were protected from powdery mildew infection, caused by *Blumeria graminis* f. Sp. *tritici*, with 250 g/ha of Quinoxyfen and 125 g/ha Bupirimate applied at 4-week intervals for 12 weeks. Individual plots were inoculated at different times as they reached heading (Zadoks 55), by spraying flag leaves with the conidial suspension to run-off. High humidity was ensured by watering the site just before inoculation and by using plastic bags secured over PVC rings (15 cm high, 30 cm diameter) to cover individual plots for 48 h after inoculation. Before being used to cover the plots, the plastic bags were misted internally with water. To shade the inoculated plants from direct sunlight, the plastic bags themselves were covered with shade-cloth bags (84–90% cover factor). At 390 °C thermal days (sum of mean daily temperatures) after inoculation, PLAD was scored on the flag leaves of five individual plants selected randomly in each plot and averaged to ensure a single numerical tan spot symptom severity value was attributed to each experimental unit.

#### Horsham, Victoria

The IWD panel was assessed at the seedling stage in the glasshouse and at the adult plant stage in an irrigated field nursery in both years. For glasshouse experiments, inoculum was prepared using a slightly modified method as described by Shankar et al. (2017). During 2015, an equal mix of six (WAC13438, 13–190, 13–198, 13–202, 14–006, 14–073) virulent isolates were used and during 2016 an equal mix of ten (Ptr15-079, Ptr15-080, Ptr15-085, Ptr15-088, Ptr15-092, Ptr15-101, Ptr15-102, Ptr15-108, Ptr15-109, Ptr15-110) were used. All isolates were obtained from the culture collection of Agriculture Victoria, Horsham. Isolates were grown on potato dextrose agar under white fluorescent and gro-lux lights at 24 ± 2 °C for 7 days. Two 3 mm^2^ plugs of each cultured isolate were then sub-cultured onto clarified V8 juice agar plates and incubated in darkness at 22 ºC for 5 days, after which the hyphal growth was flattened using a sterile metal rod. Plates were then incubated under white fluorescent and gro-lux lights at 24 °C for 24 h, and then in darkness at 16 °C for 24 h to produce conidia. Inoculum was prepared by scraping conidia from the surface of the plates using a spatula and then suspended in microfiltered sterile water. Two seeds of each line were sown into 5 cm deep pots containing potting mixture, fertiliser and trace elements. Experimental design blocks were arranged across four trays in a 2 by 2 arrangement. The experiments were conducted under natural light at 20 ± 2 ºC, and seedlings were inoculated at the two–three leaf stage (Zadoks 12–13) with the conidial suspension with concentration of ~ 3,500 spores/ml. Inoculated plants were incubated at 95–100% relative humidity in total darkness at 20 ± 1 ºC for 24 h while maintaining the same block structure and row by column layout. Inoculated seedlings were then returned to a glasshouse for 7 days to allow for symptom development. At this point, leaves that had been fully emerged at inoculation were assessed for symptom severity using a 1–9 scale in increments of 1, where 1 was low disease severity and 9 is high disease severity (Dinglasan et al. 2016). The scale uses a combination of lesion type (Lamari and Bernier 1989), lesion size, and percentage leaf area diseased relative to the response of susceptible controls. Assessed leaves symptom severity was then averaged to ensure a single tan spot symptom severity value for each of the experimental units.

Field design blocks aligned with pre-watered irrigation field bays and plants were sown in May. Each experimental unit consisted of a 50-cm row in which approximately 20 seeds were sown. Rows were 30 cm apart. At planting plots were fertilised with mono-ammonium phosphate fertiliser (70 kg/ha) treated with flutriafol fungicide (75 g /ha) to suppress stripe rust (*Puccinia striiformis*) and septoria tritici blotch (*Zymoseptoria tritici*) development. Infection was established by spreading approximately 0.5 t/ha of wheat stubble naturally infected with locally occurring Ptr from the previous year during June in both years. The infected stubble was sourced from a block of wheat planted to a variety highly susceptible to yellow spot but resistant to other important diseases. This block was also managed with flutriafol as described above. Also, during the 2016 experiment, the site received a foliar spray of a conidial suspension (~ 3,700 spores/ml) of an equal mix of eight virulent isolates (Ptr15-080, Ptr15-085, Ptr15-088, Ptr15-092, Ptr15-101, Ptr15-102, Ptr15-108, Ptr15-110). No supplementary irrigation was applied during either year as there was sufficient in-crop rainfall. Disease severity was rated using the 1–9 scale described above. During 2015, assessments were conducted when most lines were at late stem elongation (Zadoks 36) and booting (Zadoks 45), while during 2016, assessments were made when most plants were at early stem elongation (Zadoks 32), late stem elongation to early booting (Zadoks 39–41), late booting (Zadoks 49) and anthesis (Zadoks 61 to 65). Middle leaf layers were randomly assessed in most cases. For assessments made at late anthesis, the top 4 leaf layers were randomly assessed. At each assessment stage, the symptom severity across sampled leaves was averaged to ensure a single tan spot symptom severity value was attributed to each of the experimental units.

#### Toowoomba, Queensland

The IWD panel was assessed at the seedling and adult plant stages under controlled environment and in an irrigated field nursery in both years. Controlled environment and field experiments were conducted using methods described by Shankar et al. (2017). Lines were assessed in a controlled environment room at 23 ± 1 °C with each lighting fixture containing both sodium vapour and metal halide bulbs emitting PAR at 400–500 μmol m^−2^ s^−1^. Prior to seedling rating plants were grown with 14 h day and 10 h night and post-rating were switched to 20 h day and 4 h night to reduce the time to head emergence. Plants were grown in 55 mm Square Native Tube pots, 160 mm high (Garden City Plastics, Monbulk Vic), containing Searles Native Mix potting soil (Searles Pty Ltd Kilcoy QLD). Seeds were pre-germinated at 5 °C and four seeds per line were planted in each pot. Experimental design blocks were aligned with separate benches. For all the experiments conducted in Toowoomba, a mixture of ten contemporary Ptr isolates (GR2015-1, GR2015-2, GR2015-4, GR2015-5, GR2015-7, GR2015-8, GR2015-9, GR2015-10, GR2015-11, GR2015-12) was used. Inoculum was prepared as described by Shankar et al. (2017). For both seedling and adult screening, spore concentration was adjusted to 120 ± 30 spores/ml and at growth stage Zadoks 12.5 the top 2–3 seedling leaves were inoculated with 1 ml of spore suspension. Inoculated plants were incubated in the dark for about 24 h with a humidifier operating at 95–100% relative humidity for 30 min on and 30 min off. The block structure and row by column layout was maintained during the incubation period. When known susceptible and resistant varieties exhibited expected disease reactions (at around nine days after inoculation), disease was assessed on the 1–9 scale described above on the leaves that had been fully emerged at inoculation. Immediately after rating, plants were returned to the growth room and foliar and soil fertilised with half strength soluble Thrive fertiliser (Yates Australia, Padstow NSW), on a weekly basis. When individual plants had reached growth stage Zadoks 55, flag and flag minus one leaves of individual plants per pot were inoculated as above with approximately 3 ml of spore suspension and an average rating was determined 14 days later using the 1–9 scale described above The average rating ensured a single numerical tan spot symptom severity value was attributed to each experimental unit.

Lines were also sown into a field nursery under shade cloth. In 2015, the field trial layout was an RCBD with three replicates where the set of lines within each replicate was randomly allocated to positions within a 12 row by 16 column configuration. To enhance pathogen sporulation and infection, humidity was increased two or three times weekly, as necessary, at sunset using rainwater supplied micro misters for 30 to 60 min. Each plot was a 20 cm long single row with 20 cm between plots. To avoid drought stress, plants were irrigated approximately fortnightly using drip irrigation. Infection was established by spreading between rows, infected stubble from the previous year. Stubble was collected and stored over summer from plots which were inoculated in previous seasons with a suite of isolates collected to represent the variation in the region. This was augmented by inoculation during the season with isolates previously collected from the regions to represent existing variability. Impact of other diseases like rusts was minimal as trials were conducted in a series of dry years in a region where rust resistance is the highest priority. Tan spot symptom severity was assessed on a 1–9 scale when most plants were at early tillering (Zadoks 22) and anthesis (Zadoks 61–65). Whole plants were assessed at the early tillering stage while the top two layers were assessed at anthesis and averaged to ensure ratings were attributed to experimental units.

### Ptr ToxA and Ptr ToxB plant bioassays

The Ptr effector proteins Ptr ToxA and Ptr ToxB were heterologously expressed in *E. coli* SHuffle and purified using affinity chromatography (IMAC) as described by See et al. (2019). For the plant bioassays, seeds were sown in vermiculite in seedling trays, fertilised during sowing with soluble all-purpose Thrive N/P/K 25:5:8.8 (Yates Australia, Padstow NSW) at the concentration of 1 g/L and the plants were grown at 22 °C under a 12-h photoperiod in a controlled growth chamber (Conviron). Fully expanded leaves of two-week old plants were infiltrated with purified effector protein using a needleless 1 mL syringe on the adaxial surface of the leaf, at the concentration of 10 ng/µl for Ptr ToxA and 200 ng/µl for Ptr ToxB. Leaves were evaluated at 7 (Ptr ToxA) and 10 days (Ptr ToxB) post-infiltration for symptoms of Ptr ToxA-induced necrosis (presence or absence) and Ptr ToxB-induced chlorosis (See et al. 2019). Ptr ToxA-infiltrated plants were scored as either sensitive = 1 or insensitive = 0, while Ptr ToxB-induced symptoms were scored from 0 to 5 scale, with increments of 1 where 0 = no symptoms and 5 = necrosis.

### Genotyping and physical map

A 90 K custom designed Illumina SNP chip (Wang et al. 2014) was used for genotyping of the 192 IWD panel lines. The complete genetic marker set initially consisted of 51,851 unique SNP assays. This marker set was initially reduced by removing monomorphic markers and markers that contained less than 50% of observed alleles across the 192 lines. The sequences for the remaining 42,266 markers were then aligned to the *Triticum aestivum* IWGSC RefSeq V2.1 reference genome assembly (Zhu et al. 2021) with the purpose of building a physical map to be used for the downstream whole-genome analyses. To ensure high congruency between the 90 K consensus map and physical map, where possible, consensus map chromosomes were assigned to physical markers if their sequences were strongly aligned to the matching reference chromosome. For cases where the marker sequences aligned equally well on multiple genomic regions of the same chromosome, the relative consensus map position of the marker was used to choose the most appropriate physical position. Physical markers that could not be initially aligned using the consensus map were given a chromosome and physical position based on the best alignment to the reference assembly. This set of markers along with the physical map was then used to create a genetic object compatible for use in the qtl package (Broman and Wu 2019) and ASMap package (Taylor and Butler 2017) available in the R statistical computing environment (R Core Team, 2021). The functionality of these packages was then used to further diagnostically assess the quality attributes of the markers as well as the IWD lines. Specifically, it is well known the minor allele frequency (MAF) of the markers can have a dramatic impact on downstream analyses such as imputation of missing allele calls within the genetic map (Rutkoski et al. 2013) and population structure inference (Linck and Battey 2018). MAF is also known to be linked to the frequency of false positives obtained from conducting GWAS (Tabangin et al. 2009). In this research, we have removed markers if they had less than 16 observed instances of the minor allele (MAF: 8.5%) and this reduced the physical map to 27,822 polymorphic markers. Missing alleles were then imputed using the *k*-nearest neighbour (*k*-NN) algorithm (Troyanskaya et al. 2001; Rutkoski et al. 2013). To reduce the complexity of the algorithm and localise the nearest neighbours, the imputation was conducted within each chromosome individually. For each marker containing missing values, the algorithm was instructed to use its six nearest neighbours. Imputation was computationally conducted using the pedicure R package (Butler 2019). To finalise the physical map for analysis, if a group of markers were numerically equivalent, then a single marker from each group was chosen and this reduced the physical map to 20,519 unique markers.

### Tan spot phenotypic modelling

Initially, for each tan spot severity trait defined from a single row of Table 2, a linear mixed model (LMM) was used to partition and estimate genetic and non-genetic variation. For the purpose of satisfying modelling assumptions, percentage leaf area diseased traits were initially logit transformed and considered to be the new response to be analysed. Let ${\varvec{y}}=\left({y}_{1}, \dots , {y}_{n}\right)$ be a $n$ length response vector attributed to a single tan spot severity trait then the LMM had the form

1 $${\varvec{y}}={\varvec{X}}{\varvec{\tau}}+{\varvec{Z}}{\varvec{u}}+{{\varvec{Z}}}_{\mathrm{g}}{\varvec{g}}+{\varvec{e}}$$

where ${\varvec{X}}{\varvec{\tau}}$ is the fixed component of the LMM. This contained a term to capture the average expression of the trait for the different sub-populations within the IWD panel as well as average expression for each of the local controls. The fixed component of the LMM was also used to capture linear trends that may have existed across the rows or columns of the experiment. The random term ${\varvec{Z}}{\varvec{u}}$ was used to capture extraneous variation from structures associated with the design of the experiment such as benches or trays in controlled environments or complete blocks in the field. The number of the terms varied according to the type of experiment and its design. Where appropriate, terms were added to this random component of the LMM to account for broad nonlinear trends across the row or column layout of the experiment (Gilmour et al. 1997). The residual error term, ${\varvec{e}}$, was used to account for correlation between observations due to adjacency of the pots in the glasshouse, controlled environment or plots in the field experiments. We assume a more general distribution of the form ${\varvec{e}}\boldsymbol{ }\sim \boldsymbol{ }N(0,\boldsymbol{ }{\varvec{R}})$ where ${\varvec{R}}=\boldsymbol{ }{\oplus }_{i=1}^{m}$ ${{\varvec{R}}}_{i}$ was a multi-section direct sum structure with ${{\varvec{R}}}_{i}=\boldsymbol{ }\boldsymbol{ }{{\varvec{\Sigma}}}_{ic}\otimes {{\varvec{\Sigma}}}_{ir}$ containing a parameterisation for a separable AR1 × AR1 (AR1 = autoregressive process of order 1) correlation process that adequately captures the similarity of the observations across separated column and row components of the experimental design, respectively. This more general form for the residuals caters for experimental layouts that are spread across $m$ non-abutting benches in a controlled environment. For rectangular experimental layouts, $m=1$. The final term on the right hand side of (1), ${{\varvec{Z}}}_{\mathrm{g}}{\varvec{g}}$, contained a vector ${\varvec{g}}$ of length $r$ to capture the total genetic trait variation of the IWD panel sub-populations around their average expression. The distribution of the genetic effects is assumed to be ${\varvec{g}}\boldsymbol{ }\sim \boldsymbol{ }N\left(0,\boldsymbol{ }{ \sigma }_{\mathrm{g}}^{2}{{\varvec{I}}}_{r}\right)$ where ${\sigma }_{\mathrm{g}}^{2}$ is the genetic variance and ${{\varvec{I}}}_{r}$ is the identity matrix.

For each of the fitted LMMs, model residuals were found to satisfy modelling assumptions of homoscedasticity and negligible outlier influence. From each of the fitted models for the traits, empirical best linear unbiased estimators (eBLUEs) of the IWD panel sub-populations as well as empirical best linear unbiased predictions (eBLUPs) of the individual IWD panel lines were extracted for numerical and graphical summary. A generalised broad sense heritability was calculated using the formula developed by Cullis et al. (2006), namely,

$${H}^{2}=1-\frac{PE{V}_{\mathrm{a}}(\widetilde{{\varvec{g}}}, \widetilde{{\varvec{g}}})}{{2\widehat{\sigma }}_{\mathrm{g}}^{2}},$$

where $PE{V}_{\mathrm{a}}(\widetilde{{\varvec{g}}}, \widetilde{{\varvec{g}}})$ is the average pairwise prediction error variance of the eBLUPs and ${\widehat{\sigma }}_{\mathrm{g}}^{2}$ is the residual maximum likelihood (Patterson and Thompson 1971) estimate of the genetic variance for the IWD panel.

### Ptr ToxA and Ptr ToxB phenotypic modelling

Ptr ToxA-induced necrosis and Ptr ToxB-induced chlorosis were mostly consistent across the replicates of each line in the IWD panel of the plant bioassays. The lack of replicate variation indicated a single numerical value for each line may be better suited in the analysis approaches that follow. These collapsed traits could then be considered a proxy for total genetic effects of the Ptr ToxA or Ptr ToxB effectors across the population. To ensure these traits could be used in the whole-genome analyses that follow a simple effector placeholder LMM required developing. Let ${\varvec{t}}$ be a vector of collapsed Ptr ToxA or Ptr ToxB sensitivity values, then the effector baseline LMM was of the form

2 $${\varvec{t}}\boldsymbol{ }={1}_{r}\mu +{{\varvec{g}}}_{t}+{{\varvec{e}}}_{t},$$

where $\mu$ is the grand mean, ${{\varvec{g}}}_{t}$ are random total genetic effects for the IWD lines with distribution ${{\varvec{g}}}_{t}\boldsymbol{ }\sim \boldsymbol{ }N\left(0,\boldsymbol{ }{ \sigma }_{\mathrm{g}}^{2}{{\varvec{I}}}_{r}\right)$ and ${{\varvec{e}}}_{t}$ are model residuals distributed ${{\varvec{e}}}_{t}\boldsymbol{ }\sim \boldsymbol{ }N(0,\boldsymbol{ }{ \sigma }_{t}^{2}{{\varvec{I}}}_{r})$ where ${\sigma }_{t}^{2}$ is fixed at a very small positive value. This ensures all between variety variation will be appropriately attributed to ${{\varvec{g}}}_{t}$**.**

To determine the association strength of the Ptr ToxA and Ptr ToxB effectors with the complete set of tan spot severity traits defined in Table 2, the LMM defined in (1) was extended by incorporating the collapsed Ptr ToxA and Ptr ToxB sensitivity traits as centred numerical covariates into the fixed component of the model, namely

3 $${\varvec{y}}\boldsymbol{ }={{\varvec{X}}}^{\boldsymbol{*}}{{\varvec{\tau}}}^{\boldsymbol{*}}+{\varvec{Z}}{\varvec{u}}+{{\varvec{Z}}}_{\mathrm{g}}{\varvec{g}}+{\varvec{e}},$$

where ${{{\varvec{X}}}^{\boldsymbol{*}}{{\varvec{\tau}}}^{\boldsymbol{*}}={\varvec{X}}{\varvec{\tau}}+\boldsymbol{ }\Sigma }_{i=1}^{2}{{\varvec{t}}}_{i}{\gamma }_{i}$**,** and ${{\varvec{t}}}_{i}$ is the $i$ th column of ${{\varvec{Z}}}_{\mathrm{g}}{\varvec{T}}$ where ${\varvec{T}}$ is an $(r\times 2$) matrix containing the centred Ptr ToxA and Ptr ToxB covariates and ${\gamma }_{i}$ is the effect size for the $i$ th covariate. All other terms have been defined previously. This extended model will be referred to as the baseline LMM for an individual tan spot severity trait. From each of the fitted trait models, estimates of the Ptr ToxA and Ptr ToxB effects and their standard errors were extracted and the significance of the association with the tan spot trait was summarised using a Logarithm of Odds (LOD) scores derived from a simple asymptotic Taylor series expansion of a likelihood function with covariate parameter, ${\gamma }_{i}$ say, expanded around zero, namely,

4 $$LO{D}_{{\gamma }_{i}}= \frac{1}{2}{\mathrm{log}}_{10}[\mathrm{exp}({\gamma }_{i}^{2}/{\sigma }_{{\gamma }_{i}}^{2})]$$

where ${\sigma }_{{\gamma }_{i}}^{2}$ is the variance of the covariate effect. In practice, estimates ${\widehat{\gamma }}_{i}$ and ${\widehat{\sigma} }_{{\widehat{\gamma }}_{i}}^{2}$ are extracted from the model results to form the empirical LOD scores. An identical approach to calculation of LOD scores occurs in the whole-genome average interval mapping (WGAIM) software (Taylor and Verbyla 2011) discussed in more detail in the next section.

### One-step whole-genome analyses

Let ${\varvec{M}}$ be an $\left(r\times m\right)$ matrix of whole-genome marker-based information for the IWD panel. To conduct an efficient one-step whole-genome analysis of each tan spot severity trait and effector trait, a modified whole-genome average interval mapping (WGAIM) approach (Verbyla et al. 2007, 2012) was adopted. In what follows, we focus on developing the extension for the baseline tan spot severity LMM in (3) with recognition this extension also applies, without loss of generality, to the simplified effector baseline LMM in (2). This required extending the LMMs defined by (3) by considering a partition of the random total genetic effects, namely

5 $${\varvec{g}}={\varvec{a}}+{\varvec{p}},$$

where ${\varvec{a}}$ is a vector of additive genetic line effects that are distributed ${\varvec{a}}\boldsymbol{ }\sim \boldsymbol{ }N(0,\boldsymbol{ }{\sigma }_{\mathrm{a}}^{2}{{\varvec{G}}}_{\mathrm{a}}/c)$ with ${{\varvec{G}}}_{\mathrm{a}}={\varvec{M}}{{\varvec{M}}}^{T}$ representing an $\left(r\times r\right)$ additive relationship matrix reflecting the marker based relationships between the lines and $c=\mathrm{trace}({{\varvec{G}}}_{\mathrm{a}})/r$ (Forni et al. 2011). The final term on the right-hand side was the residual or non-additive polygenic effects and were assumed to have a distribution ${\varvec{p}}\boldsymbol{ }\sim \boldsymbol{ }N\left(0,\boldsymbol{ }\boldsymbol{ }{\sigma }_{\mathrm{p}}^{2}{{\varvec{I}}}_{r}\right)$**.**

Substituting (5) into (3) the initial whole-genome analysis LMM becomes

6 $${\varvec{y}}={{\varvec{X}}}^{\boldsymbol{*}}{{\varvec{\tau}}}^{\boldsymbol{*}}+{\varvec{Z}}{\varvec{u}}+{{\varvec{Z}}}_{\mathrm{g}}{\varvec{a}}+{{\varvec{Z}}}_{\mathrm{g}}{\varvec{p}}+\boldsymbol{ }{\varvec{e}}.$$

Note the inclusion of the term ${{\varvec{X}}}^{\boldsymbol{*}}{{\varvec{\tau}}}^{\boldsymbol{*}}$ ensures the Ptr ToxA and Ptr ToxB scores for the IWD panel are included as covariates to nullify their associated genomic effect in the whole-genome analyses. After this initial fit, the significance of the additive genetic variance, ${\sigma }_{\mathrm{a}}^{2}$ is assessed using a simple likelihood ratio test (LRT) between LMMs (6) and (3). If significant at an alpha level of 0.05, the BLUPs of the additive genetic effects, $\widetilde{{\varvec{a}}}$, were extracted from the fitted LMM of (6) and the predicted marker effects and their variances are calculated through back transformation (Norman et al. 2017) using

$$\widetilde{{\varvec{q}}}=\boldsymbol{ }{{\varvec{M}}}^{T}{{\varvec{G}}}_{\mathrm{a}}^{-1}\widetilde{{\varvec{a}}}/c$$

7 $$\mathrm{var}(\widetilde{{\varvec{q}}})=\boldsymbol{ }{{\varvec{M}}}^{T}{{\varvec{G}}}_{\mathrm{a}}^{-1}({\sigma }_{\mathrm{a}}^{2}{{\varvec{G}}}_{\mathrm{a}}/c\boldsymbol{ }-PEV(\widetilde{{\varvec{a}}},\widetilde{{\varvec{a}}})){{\varvec{G}}}_{\mathrm{a}}^{-1}{\varvec{M}}/{c}^{2}$$

where $PEV\left(\widetilde{{\varvec{a}}},\widetilde{{\varvec{a}}}\right)$ is the prediction error variance of the additive genetic line effects. Marker-based outlier statistics (Verbyla et. al, 2007) are then calculated for the $j$ th marker as ${\widetilde{q}}_{j}^{2}/\mathrm{var}({\widetilde{q}}_{j})$ where ${\widetilde{q}}_{j}$ is the predicted effect of the $j$ th marker and $\mathrm{var}({\widetilde{q}}_{j})$ is the $j$ th diagonal element of (7). The marker with the largest outlier statistic across the whole genome is then said to be linked to a putative QTL. This marker is then removed from ***M*** in the model and placed as an additive random covariate in the baseline LMM (3) as well as the whole-genome analysis LMM (6). Both models are re-estimated and the process of finding a significant marker is then repeated. An informative flow diagram of how the WGAIM algorithm proceeds can be found in Verbyla et al. (2012). This process is halted if the LRT of the additive variance parameter, ${\sigma }_{\mathrm{a}}^{2}$, is found to be non-significant. For $t$ putative QTL, the final LMM is then

$${\varvec{y}}={{\varvec{X}}}^{\boldsymbol{*}}{{\varvec{\tau}}}^{\boldsymbol{*}}+ \sum\limits_{{i = 1}}^{t} {\varvec{m}_{i} q_{i} + \varvec{Zu} + \varvec{Z}_{{\text{g}}} \varvec{a}_{{ - t}} + \varvec{Z}_{{\text{g}}} \varvec{p} + \varvec{e}}$$

where ${{\varvec{m}}}_{i}$ is the column of ${{\varvec{Z}}}_{\mathrm{g}}{\varvec{M}}$ associated with the $i$ th selected marker and ${{\varvec{a}}}_{-t}\boldsymbol{ }\sim \boldsymbol{ }N(0,\boldsymbol{ }{ \sigma }_{\mathrm{a}}^{2}{{\varvec{M}}}_{-t}{{\varvec{M}}}_{-t}^{T}/c)$ where ${{\varvec{M}}}_{-t}$ is the marker matrix ${\varvec{M}}$ with the $t$ markers removed. The retaining of the marker based additive relationship matrix containing markers not selected by the algorithm ensures selected marker effects are tested at the appropriate level in the structural hierarchy of the final LMM. Each of the markers selected is then summarised with their effect size and LOD score defined in (4), as well as an approximate contribution of each marker to the overall genetic variance. Further details of these calculations can be found in Verbyla et al. (2012). To understand the pairwise linkage disequilibrium between detected markers, the Pearson’s correlation was used and graphical summaries were presented using the R package corrplot (Wei and Simko 2021).

### Tan spot genomic prediction

For each of the tan spot severity traits, we used genomic prediction to assess the relative total genetic performance of the lines under disease pressure from Ptr. The genomic prediction was conducted using the initial one-step whole-genome analysis LMM defined in (6) which is similarly defined in Norman et al. (2017). Key elements of this genomic prediction LMM include the incorporation of terms to account for extraneous non-genetic sources of variation. Additionally, the inclusion of Ptr ToxA and Ptr ToxB covariates ensured their genomic effects are removed and the prediction is focusing on the cumulative effects from the remaining genomic regions. Lastly, model (6) contains a partitioning of the total genetic effects using (5) that incorporate additive line genetic effects, ${\varvec{a}}$**,** from a whole-genome marker-based relationship matrix and non-additive residual genetic effects, ${\varvec{p}}$. In most genomic prediction studies, the focus is on accurately predicting a breeding value based on additive genetic effects only. In this research, we used the genomic prediction

$${\widetilde{g}}_{ij}={\widehat{\mu }}_{i}+{\widetilde{a}}_{ij}+\boldsymbol{ }{\widetilde{p}}_{ij}$$

where ${\widehat{\mu }}_{i}$ is the eBLUE of the $i$ th sub-population and ${\widetilde{a}}_{ij}$ and ${\widetilde{p}}_{ij}$ are the eBLUPs of the additive and non-additive genetic effects for the $j$ th line within the $i$ th sub-population. This calculation was preferred in this research as it predicts the total genetic value of the lines across the different sub-populations, providing the most accurate representation of the genetic resistance of the lines to Ptr. To calculate prediction accuracy we used the model based formula derived in Mrode (2014) where, for the $j$ th line within the $i$ th sub-population, the reliability of the prediction is calculated using

$${\rho }_{ij}=1- PEV{\left(\widetilde{{\varvec{g}}},\widetilde{{\varvec{g}}}\right)}_{ij}/{{\varvec{\Omega}}}_{ij}$$

where $PEV{\left(\widetilde{{\varvec{g}}},\widetilde{{\varvec{g}}}\right)}_{ij}$ is the prediction error variance of the $j$ th line within the $i$ th sub-population and ${{\varvec{\Omega}}}_{ij}$ is the associated diagonal element of ${\varvec{\Omega}}={\sigma }_{\mathrm{a}}^{2}{{\varvec{G}}}_{\mathrm{a}}/c +{\sigma }_{p}^{2}{{\varvec{I}}}_{r}$. The overall genomic prediction accuracy is the square root of $\rho = {\sum }_{ij}{\rho }_{ij}$/*r*. This method closely matches method 7 outlined in Estaghvirou et al. (2013) and provides an approach to calculating genomic prediction accuracy that does not require cross-validation.

### Computations

All phenotypic models discussed in this research were fitted using version 4 of the linear mixed modelling software ASReml-R (Butler et al. 2018) bundled as an R package for use in the R statistical computing environment (R Core Team, 2021). The ASReml-R package contains a suite of functionality for fitting and diagnosing complex LMMs and uses the residual maximum likelihood (REML) algorithm of Patterson and Thompson (1971) to estimate model parameters. The package is available to download from VSN International (https://www.vsni.co.uk/). Diagnostic assessment of models was conducted using ASReml-R functions as well as functions from the linear mixed modelling post-processing package ASExtras available for download from https://mmade.org/. All whole-genome analyses were conducted using minor modifications of the WGAIM V2 R package (Taylor and Verbyla 2011) available in the R statistical computing environment. The WGAIM package depends on ASReml-R for all its LMM fitting, and this requires a licensed version of the ASReml-R software to be installed.

## Results

### Physical map

A graphical and tabular summary of the 20,519 genetic markers aligned to the physical map is provided in Supplementary Figure S1. The figure indicates a tight density of markers for most A and B chromosomes. As expected, the marker density was reduced for the D chromosomes, with reduced density across the centromeric regions of each of the chromosomes. The physical alignments of the markers were also assessed against the 90 K consensus map derived in Wang et al. (2014) (Supplementary Figure S2.). The figure indicates a strong monotonic relationship between the consensus map positions and the physical positions of the markers within each of the chromosomes. The strength of these relationships was reduced amongst some of the D chromosomes.

### Tan spot phenotypic analyses

**Fig. 1:**
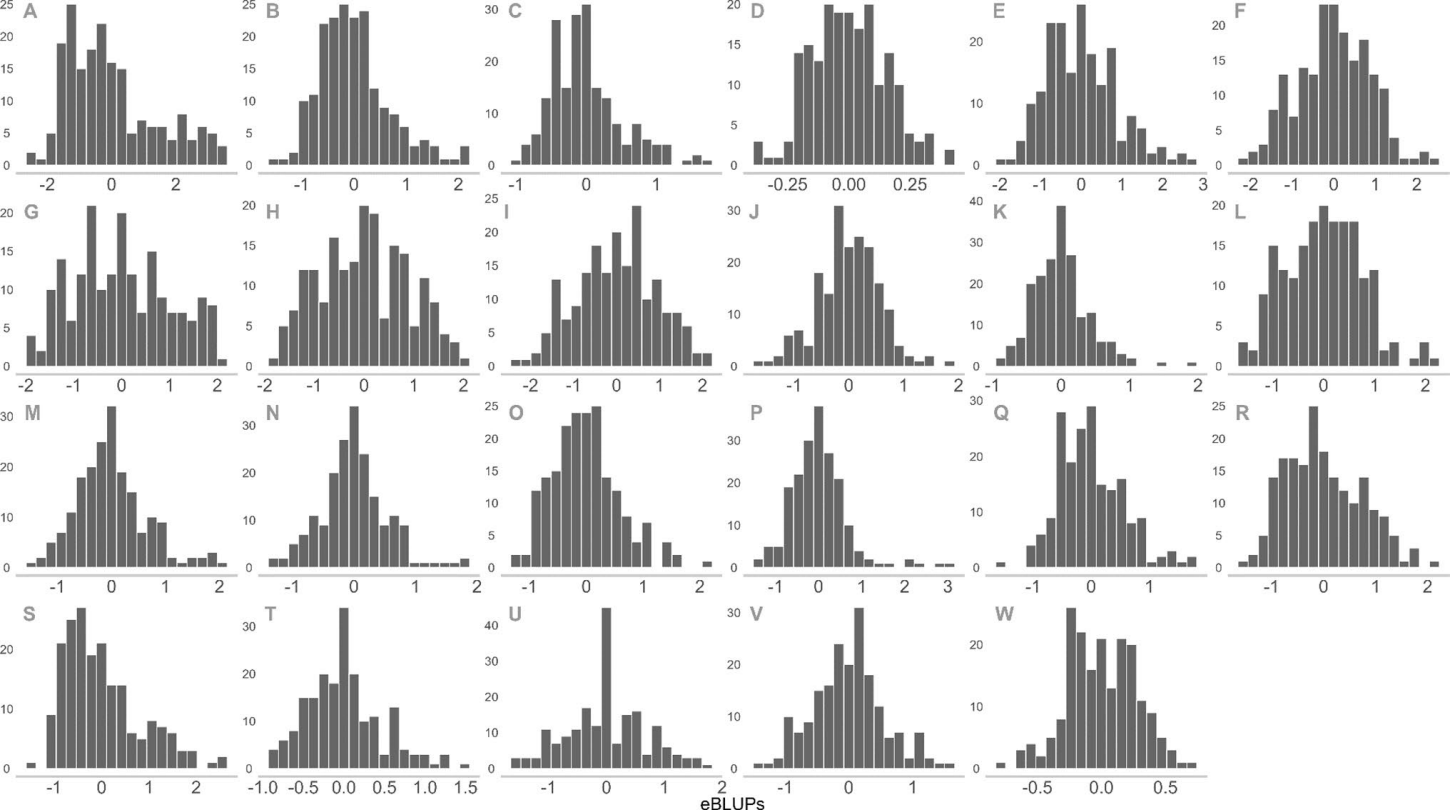
Graphical summary of counts (*y*-axis) of the empirical best linear unbiased predictions (eBLUPs) on the *x*-axis extracted from each phenotypic model for tan spot severity of the international wheat diversity lines. Each panel represents a single tan spot severity trait identified by the alphanumeric code in the top left hand corner (see Table 2). eBLUPs for flag leaf traits are presented on the scale of the transformation

Generalised broad sense heritability ranged from 0.123 to 0.899 (Table 2). Heritabilities calculated from South Perth controlled and field experiments were consistently higher than those from the other two locations. For the seedling and adult tan spot traits in the controlled environment and field experiments at Toowoomba in 2016, a lower heritability was observed. This reduced heritability aligns with the lower number of IWD line replicates used in these experiments. For each of the tan spot severity trait, the genetic eBLUPs are graphically displayed as histograms in Fig. 1. For average flag leaf tan spot severity traits (K, L, N, O), the eBLUPs are represented on the scale of the logit transformation. There was transgressive segregation for all tan spot severity traits. In the 2015 Horsham and Toowoomba controlled environment and field experiments (A, B, C, P, Q, R, S), the eBLUPs indicate there was a slight skewness towards reduced disease severity across the three sub-populations.

**Fig. 2:**
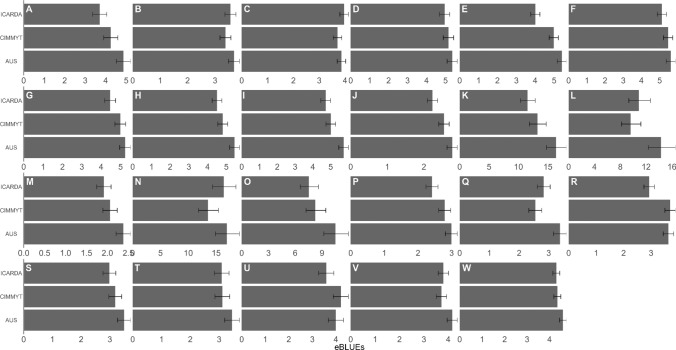
Summary of the empirical best linear unbiased estimators (eBLUEs) of the sub-population types extracted from each tan spot severity model. Each panel represents a single tan spot severity trait identified by the alphanumeric code in the top left hand corner (see Table 2). The error bars represent half-LSDs where non-overlapping error bars within each panel indicate significantly different tan spot severity between sub-population types

The eBLUEs of tan spot severity for each of the sub-populations varied depending on the trait and the location where the experiment was conducted (Fig. 2). At Horsham with the exception of tan spot symptom severity traits measured from the field experiment in 2015 (B, C), the glasshouse experiment in 2016 (D) and at early booting in the 2016 field experiment (F), on average ICARDA lines exhibited significantly reduced symptoms compared to the AUS lines (E, G, H, I). For tan spot severity traits assessed at early and late anthesis at Horsham 2016 (H, I), the CIMMYT lines also showed significantly reduced disease symptoms compared to the AUS lines. The average tan spot severity of ICARDA wheat lines was significantly less than AUS wheat lines in all traits measured at South Perth experiments (J-O) except for the traits from the 2015 field experiment and the 2016 controlled environment adult experiment (L and N), where tan spot was assessed at heading. The CIMMYT lines also showed significantly reduced severity compared to AUS lines in the traits assessed at the two 2015 South Perth adult tan spot experiments (K and L). Compared to AUS wheat lines, ICARDA lines showed significantly reduced disease severity in all the traits measured at 2015 Toowoomba controlled environment and field experiments (P-S). A significant reduction in tan spot severity was also observed from the CIMMYT lines at the adult stage of assessment in the Toowoomba 2015 controlled environment (Q) as well as the seedling stage of assessment in the Toowoomba 2016 field experiment (V).

### Ptr effector bioassay analyses

After collapsing of replicate data to generate the Ptr ToxA, Ptr ToxB sensitivity traits, 28% of IWD lines were found to be sensitive to the Ptr ToxA effector with 24% of lines were sensitive to Ptr ToxB with varying degree of sensitivity/chlorosis (scores of 0.5 to 2) observed. A whole-genome analysis was performed for the Ptr ToxA and Ptr ToxB sensitivity traits scored across the IWD panel, and a Manhattan plot of the QTL results is given Supplementary Figure S3. As expected, for Ptr ToxA the results indicated near 100% of the genetic variation was attributed to a single locus on the long arm of 5B linked to “BS00010590_51”, the 764^th^ marker in the physical position 549.79 Mb. Similarly, the results for Ptr ToxB indicated a strong association to the 179^th^ marker “BS00072619_51” on the short arm of 2B in the physical position 27.13 Mb.

**Fig. 3:**
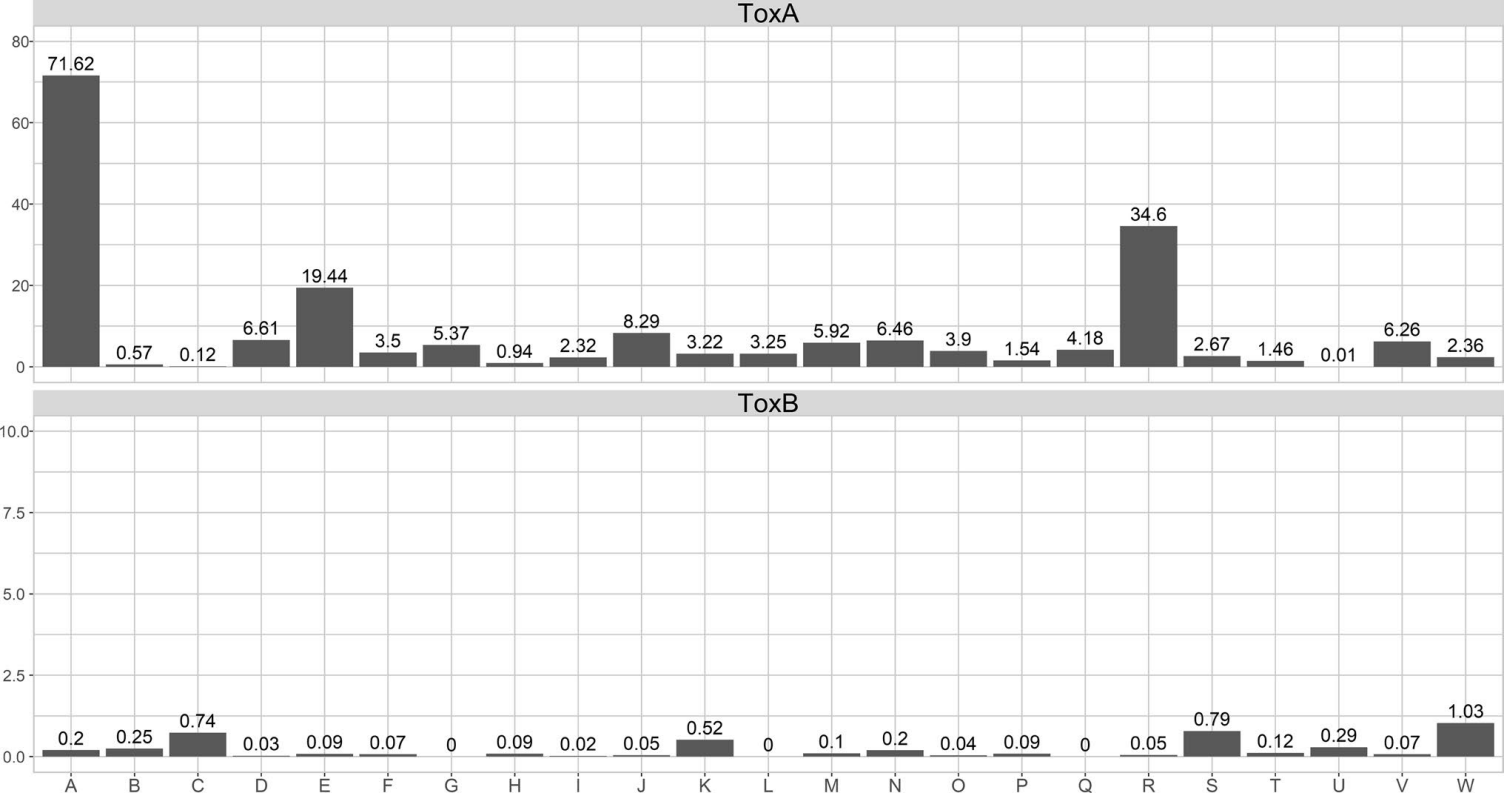
LOD scores for the association of pure ToxA and ToxB effectors with tan spot traits defined in Table 2. To maximise interpretation, numerical versions of LOD scores have been included at the top of bars

The strength of the association between wheat Ptr ToxA and Ptr ToxB sensitivity and the complete list of tan spot severity traits defined in Table 2 is given in Fig. 3. A very significant association (*p*-value < 0.0001; LOD = 71.62) occurred for Prt ToxA with seedling tan spot severity assessed at the seedling stage in the glasshouse at Horsham 2015 (A), with only smaller associations for other traits from the same year and location (B and C). In Horsham 2016, a very significant association (*p*-value < 0.0001; LOD = 19.44) was found for Ptr ToxA and tan spot severity measured at early stem elongation (E) and, interestingly, the strength of this association was dramatically reduced with the remaining tan spot traits measured at later stages of the plants’ growth in the same experiment (F-I). In South Perth, the strongest associations with Ptr ToxA sensitivity occurred with the tan spot severity trait measured at the seedling stage of the 2015 experiment (J) and the tan spot traits measured at the seedling and adult stages of the field and controlled environment experiments in 2016 (M, N, O). Other traits obtained from South Perth controlled environments exhibited much weaker association. Tan spot severity assessed at seedling stage of the 2015 field experiment of Toowoomba (R) had the strongest association with Ptr ToxA (*p*-value < 0.0001; LOD = 34.6), with other tan spot traits from Toowoomba in 2015 and 2016 having much weaker or no association. In contrast to Ptr ToxA association results, the bottom panel of Fig. 3 indicates there was only minimal or negligible association between wheat Ptr ToxB sensitivity and tan spot severity traits across all location, years, environment types and plant development assessment stages.

### Tan spot whole-genome analysis

**Fig. 4:**
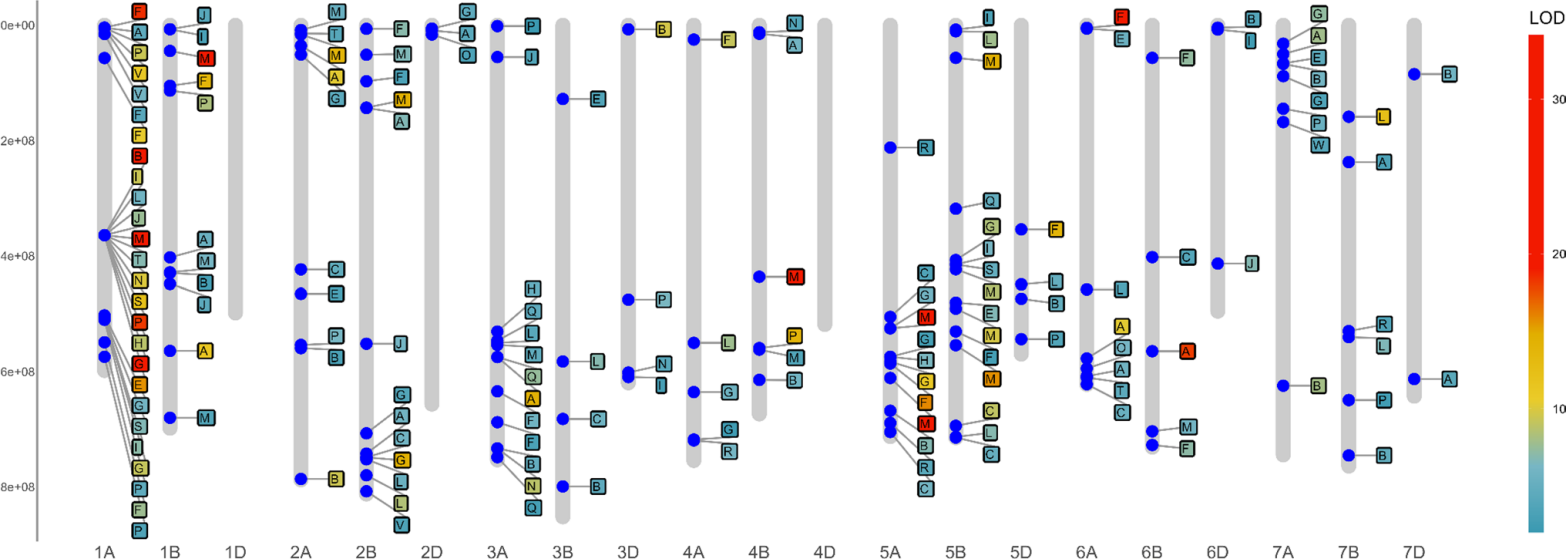
Linkage map detailing the physical location (Mbp) of the 158 QTL (see Supplementary Table S2 for further details) detected for the complete set of tan spot severity traits defined in Table 2. Boxes around trait codes are shaded according to their LOD score. LOD scores shading was capped at 25 to ensure there is differentiation at the lower end of the scale

Whole-genome analysis of the complete set of tan spot severity traits defined in Table 2 detected 158 significant marker associations at the familywise alpha level 0.05 across the 21 wheat chromosomes (Supplementary Table S2). Table S2 indicates there were 31 putative QTL with LOD scores exceeding 10 with 8 of these QTL with LOD scores greater than 20. An additional graphical summary of these results is displayed in Fig. 4 and presents the physical linkage map overlaid with QTL. We have considered groups of markers associations to be linked to the same putative QTL if they are in strong linkage disequilibrium (LD) on the same chromosome. To assess this, a graphical representation of the LD between significant markers from Supplementary Table S2 is given in Supplementary Figure S4 (a–e). It is clear from these results that there is a distinct lack of repeatability of the QTL with more than 120 QTL being classed as singletons.

Here, we have focussed on repeatability of QTL where markers in LD have an association with at least three different tan spot severity traits (Table 3). To identify short- and long-arm chromosomal regions, we used the centromeric delimiter data provided in Appels et al. (2018). Table 3 indicates that a large selection of tan spot traits assessed at various plant development stages were associated with a highly significant QTL on the short arm of 1A (TQTL-1A.1). Closer inspection of Table S2 indicates the majority of the traits were associated with a physical marker located at 364 Mb on RefSeq V2.1. However, the consensus map position indicates this marker is most likely located on 1AS near 20 cM with additional evidence from physical markers in close LD at the start of 1A (Supplementary Figure S4). For the five tan spot traits temporally measured in the field at Horsham in 2016 (E-I), the TQTL-1A.1 effect size varies suggesting that the expression of the QTL potentially changes during plant development (Table 3, Table S2). For five tan spot traits, a second significant QTL on the long arm of 1A (TQTL-1A.2) was discovered at 502–510 Mb and appears to be mostly associated with assessment of tan spot at later stages of plant development (Table 3). Three tan spot traits, with one seedling trait exhibiting a highly significant QTL (LOD > 30), were found on the short arm of 1B (TQTL-1B.1). The consensus map suggests this highly significant QTL could be on 1A, but there is no additional LD evidence that this marker or other markers in the group are in close LD with significant markers linked to TQTL-1A.1 and TQTL-1A.2 (Supplementary Figure S4). A QTL on 4BL (TQTL-4B.1) was found to be associated with three tan spot traits assessed at the seedling and booting stage of plant development (Table 3). A broad collection of marker associations was discovered across 5B, with a significant QTL on 5BL (TQTL-5B.1) associated with tan spot severity in the adult stages of plant development (Table 3). As expected, the inclusion of Ptr ToxA as a genetic covariate in the whole-genome analyses models ensured there were minimal associations from markers in LD with the Ptr ToxA physical marker detected at 549 Mb on 5BL.

**Table 3:** Summary of significant QTL detected from whole-genome analysis of tan spot traits detected for at least three traits (see Supplementary Table S2 for full list of QTL)

QTL name	Phys. interval (Mbp)	Cons. interval (cM)	Ave. LOD	Ave. %GV	Trait codes
TQTL-1A.1	4.0–364.1	13.73–20.29	13.55	19.17	B, E, F, G, H, I, J, L,
					M, N, P, S, T, V
TQTL-1A.2	502.4–510.2	66.31–85.69	7.34	10.98	F, G, I, P, S
TQTL-1B.1	6.9–44.5	8.36–53.61	12.83	8.27	I, J, M
TQTL-4B.1	559.2–614.4	71.29–76.07	7.78	9.50	B, P, M
TQTL-5B.1	318.3–414.7	40.55–48.29	6.15	12.50	G, I, Q, S

This includes physical map distance interval (Phys. Interval), consensus map interval (Cons. Interval), average LOD score (Ave. LOD), average per cent of genetic variation (Ave. %GV). Tan spot trait codes are given in the final column (see Table 2)

#### Tan spot genomic prediction

**Fig. 5:**
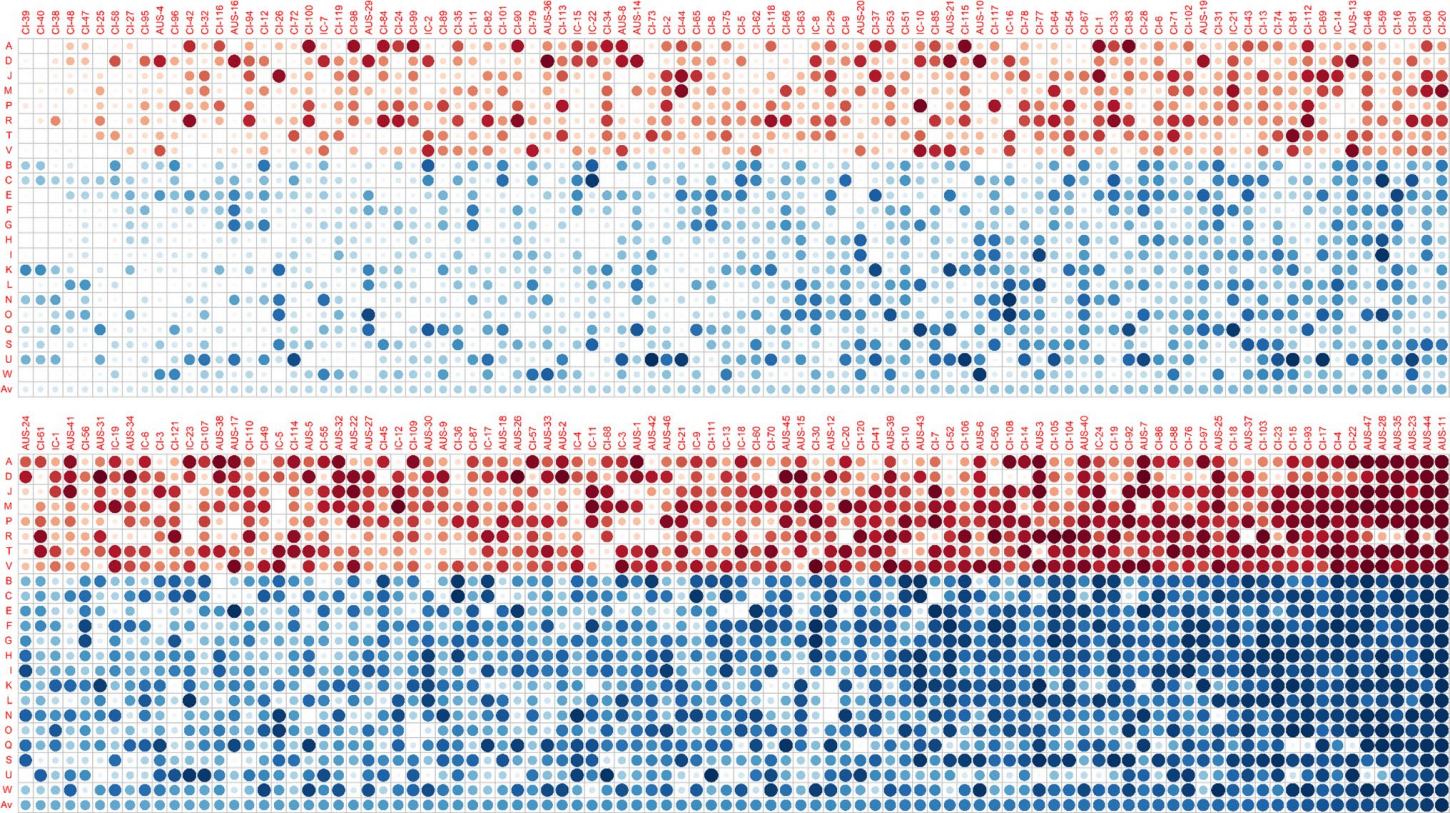
A visual heat map of the predicted tan spot severity rankings for the 192 varieties of the international wheat diversity panel (columns) for each of the tan spot severity traits (rows) defined in Table 2. For any given trait, smaller and more transparent dots represent reduced tan spot severity relative to the rest of the population and larger more opaque dots represent higher tan spot severity. The tan spot traits are partitioned into two distinct sections with traits assessed at seedling stage in red and traits assessed at adult stage in blue. The varieties are ordered by average rankings across all tan spot traits given in the bottom row of the heat map (colour figure online)

For each tan spot trait, the eBLUEs of the sub-populations were added to the additive eBLUPs and non-additive eBLUPs to form total genetic predictions of the each of the lines in the IWD panel. For all traits except tan spot severity in Horsham 2016 assessed at the seedling stage (D), genomic prediction accuracies indicate a strong influence from the genetics (Supplementary Figure S5) and this aligns with the high heritabilities obtained from the phenotypic modelling. As the scoring system differed between traits and some traits were transformed for analysis purposes, the genomic prediction variety rankings were used to provide a standardisation of the relative resistance of each variety within a trait. To visualise this resistance, rankings for each variety were averaged across the tan spot traits and varieties were ordered from most resistant to most susceptible by the average ranking (Fig. 5). In the figure, traits were also ordered to provide a distinct partitioning between seedling and adult tan spot severity assessment. As expected, this process revealed many ICARDA lines with excellent genetic resistance to tan spot, including IC-7, IC-2, IC-15 and IC-22. Within the Australian sub-population, AUS-4, AUS-16, AUS-29 and AUS-36 also showed broad tan spot genetic resistance across all seedling and adult assessed traits. However, the lines predicted to be most resistant across all tan spot severity traits were in the large CIMMYT sub-population, including CI-25, CI-27, CI-32, CI-38, CI-39, CI-40, CI-42, CI-47, CI-48, CI-58, CI-95 and CI-96. Interestingly, the top five CIMMYT lines CI-38, CI-39, CI-40, CI-47 and CI-48 all exhibited the greatest genetic resistance at the seedling stage and showed some mild susceptibility when plants were at various adult stages of development. The presentation of the traits in Fig. 5 has allowed visual identification of lines that appear to be more genetically resistant at either seedling or adult stages of plant development. Some examples of lines that show greater genetic resistance at adult compared to seedling stage of plant development include CI-1, CI-2, CI-98, CI-99, CI-112 and CI-113. Lines exhibiting greater resistance in the seedling stage compared to the adult stage are AUS-10, CI-51, CI-67 and CI-59.

## Discussion

In this research, we have conducted thorough phenotypic and genotypic experimentation on tan spot severity in an internationally diverse set of wheat lines using Australian Ptr isolates. Similar to Shankar et al. (2017), experiments were conducted in three locations across Australia under controlled environment and field conditions, and the experiments were repeated across two years. Plants were mostly inoculated at the seedling stage with a combination of Ptr isolates, and tan spot symptom severity was assessed visually (see Table 2). All tan spot severity traits were analysed using an LMM that partitioned genetic and non-genetic variation. Strong heritability was exhibited by nearly all tan spot severity traits. Within the sub-populations used in this IWD panel, the ICARDA lines identified by Shankar et al. (2015) and used here, had almost universally greater average resistance to Australian Ptr isolates than the CIMMYT and AUS sub-populations.

The IWD panel was also screened for sensitivity to Ptr ToxA and Ptr ToxB using purified effector proteins in plant bioassays and assessed for necrosis and chlorosis. A GWAS was then conducted and Ptr ToxA sensitivity aligned with a single locus on the long arm of 5B conferring its definitive link to *Tsn1* (Faris 1996; Effertz et al. 2002). The importance of the ToxA-*Tsn1* interaction during seedling responses to tan spot has been demonstrated in a panel of 40 commercial Australian wheat varieties (See et al. 2018) and 257 wheat accessions from the Vavilov collections (Dinglasan et al. 2018). The IWD panel only contains 10 lines in common with the lines used in See et al. (2018) so it unfortunately cannot be used for substantive comparisons. However, our research does confer a strong interaction with Ptr ToxA during seedling responses to tan spot. Similarly, in the IWD panel, Ptr ToxB sensitivity was strongly linked to a single locus on the short arm of 2B within the physical map region for *Tsc2* defined by Corsi et al. (2020). The inclusion of Ptr ToxA and Ptr ToxB covariates in the tan spot phenotypic models validated the ubiquitous presence of the Ptr ToxA effector in Australian Ptr isolates (Faris and Friesen 2005). However, high association of Ptr ToxA sensitivity with tan spot disease severity was mainly observed at the seedling stage of plant development in both field and glasshouse experiments but not at the later growth stages. This is in agreement with a recent study in which Ptr ToxA sensitive wheat lines derived from a bi-parental recombinant inbred line (RIL) population exhibited tan spot resistance at the adult growth stage (Dinglasan et al. 2021). Similar to other crop diseases, it is also possible quantitative resistance is governed by many genes with small effects that are not associated with effector interactions (Cowger and Brown 2019). The universal non-significance of the Ptr ToxB covariate for all tan spot traits also aligns with previously reported studies on the lack of Ptr ToxB effector presence in Australian Ptr isolates (See et al. 2021).

We used a one-step whole-genome analysis approach for detection and selection of significant tan spot related QTL. It used an extended LMM to partition and estimate the complex genetic and non-genetic variation arising in each of the tan spot experiments and consequently avoided the need for two stage analysis approaches commonly implemented in QTL and GWAS-related software packages (Bradbury et al. 2007; Tang et al. 2016; Broman and Wu 2019). This method also circumvents the computational requirement to individually scan the complete set of 20 K + unique markers as well as determine an appropriate familywise error rate to use as a threshold for multiple comparisons. With this approach we detected 158 marker associations across the 21 wheat chromosomes with 31 putative QTL with a LOD score exceeding 10. No marker associations were detected for tan spot severity traits assessed at the seedling stage of plant development in Horsham in 2016 and the adult stage of plant development in the controlled environments of South Perth 2015 and Toowoomba 2016. For nearly all tan spot traits a very significant QTL was found on 1AS between 13 and 21 cM. This has previously been identified in many other tan spot genetic analyses (Singh et al. 2016; Shankar et al. 2017; Liu et al. 2020) and is most likely co-localised with the Ptr ToxC sensitivity gene, *Tsc1* (Effertz et al. 2002). Interestingly, the physical marker for this QTL was located on the long arm of 1A at 362 Mbp in RefSeq v2.1 and previous RefSeq versions of 1A pseudo-molecules. However, the marker displayed significant linkage disequilibrium with other markers on 1AS (see Supp Figure S4 a). A QTL was found on 1AL and was highly suggestive of a secondary interaction of Ptr in adult plants in the later stages of development. A similar genomic region was tentatively reported in Dinglasan et al. (2019) where plants were also assessed at a later stage of development. A moderate size QTL was found on 1BS and this has been reported in other studies including Shankar et al. (2017) and (Liu et al. 2020). A QTL associated with three tan spot traits was found on the short arm of 4B, and this was also reported in Shankar et al. (2017) with a further two similar closely linked regions reported in Dinglasan et al. (2019). A broad collection of marker associations were found on 5B and many of these genomic regions have been tentatively reported in Liu et al. (2020). This current study now provides evidence of a strongly significant QTL on 5BL between 318 and 414 Mb that appears to be not co-localised with the ToxA sensitivity gene *Tsn1*.

The results of the whole-genome analysis indicated there were more than 120 non-repeatable QTL across 19 chromosomes of the wheat genome. This indicates the potentially highly polygenic nature of tan spot severity measurements obtained from experiments conducted under varying trial management constraints such as varied inoculation methods, controlled or field experimentation and assessment of disease at various plant development stages. To address this trait polygenicity, we used a one-step genomic prediction model based on genomic BLUP (Norman et al. 2017; 2018) as this was shown to perform well against other genomic prediction modelling approaches (Muqaddasi et al. 2021) where tan spot severity was analysed. Our genomic prediction accuracies were substantially higher than Muqaddasi et al. (2021) indicating a strong genetic influence in the tan spot severity traits suggesting the relative predictions are ideal for selecting lines for resistance and susceptibility. Although Singh et al. (2016) and Phuke et al. (2020) identified some sources of Ptr resistance in CIMMYT populations, this research has definitively identified multiple CIMMYT lines with broad resistance to Australian based Ptr that also exhibited better resistance than current Australian elite germplasm. Given these positive results, we are now exploring the possibility of more complex genomic prediction modelling approaches, such as repeated measures or multi-environment genomic prediction (Tolhurst et al. 2019), that may provide further insight into the trait polygenicity.

In summary, we have assembled an international wheat diversity (IWD) panel with broad levels of tan spot resistance and then conducted extensive phenotypic and genotypic analyses of tan spot severity traits with Australian Ptr isolates, with assessment over two years and multiple locations. Phenotypic analyses revealed strong heritability of tan spot severity traits, with ICARDA lines on average showing reduced tan spot symptoms compared to other sub-populations. One-step whole-genome analyses detected a large QTL on 1AS that is most likely co-locating with the ToxC sensitivity gene *Tsc1*. The lack of repeatable QTL prompted us to conduct a genomic prediction model where multiple CIMMYT lines were found with broad genetic resistance to Australian Ptr. These lines will provide an invaluable resource for Australian wheat plant breeding programmes to improve tan spot resistance of future varieties.

## Supplementary Information

Below is the link to the electronic supplementary material.

Supplementary file2 (DOCX 1359 KB)

Supplementary file3 (CSV 6 KB)

Supplementary file1 (CSV 14 KB)

## Data Availability

The datasets generated during and/or analysed in this research are available in the Figshare repository, https://figshare.com/s/aa81d4b68755bef1b77f. (Currently under temporary embargo as IP constraints are finalised but will be publicly available before publication).
