# Supplementary material for: An international wheat diversity panel reveals novel sources of genetic resistance to tan spot in Australia
Source: Theor Appl Genet. 2023 Mar 13;136(3):61. doi: 10.1007/s00122-023-04332-y (PMC10011302; doi:10.1007/s00122-023-04332-y)
Supplement: Supplementary file 1 — Supplementary file2 (DOCX 1359 KB) [file 122_2023_4332_MOESM1_ESM.docx]

**Fig. S1** Physical map and tabular summary of the 20,519 polymorphic markers for the international wheat diversity panel selected from the 90K SNP chip.


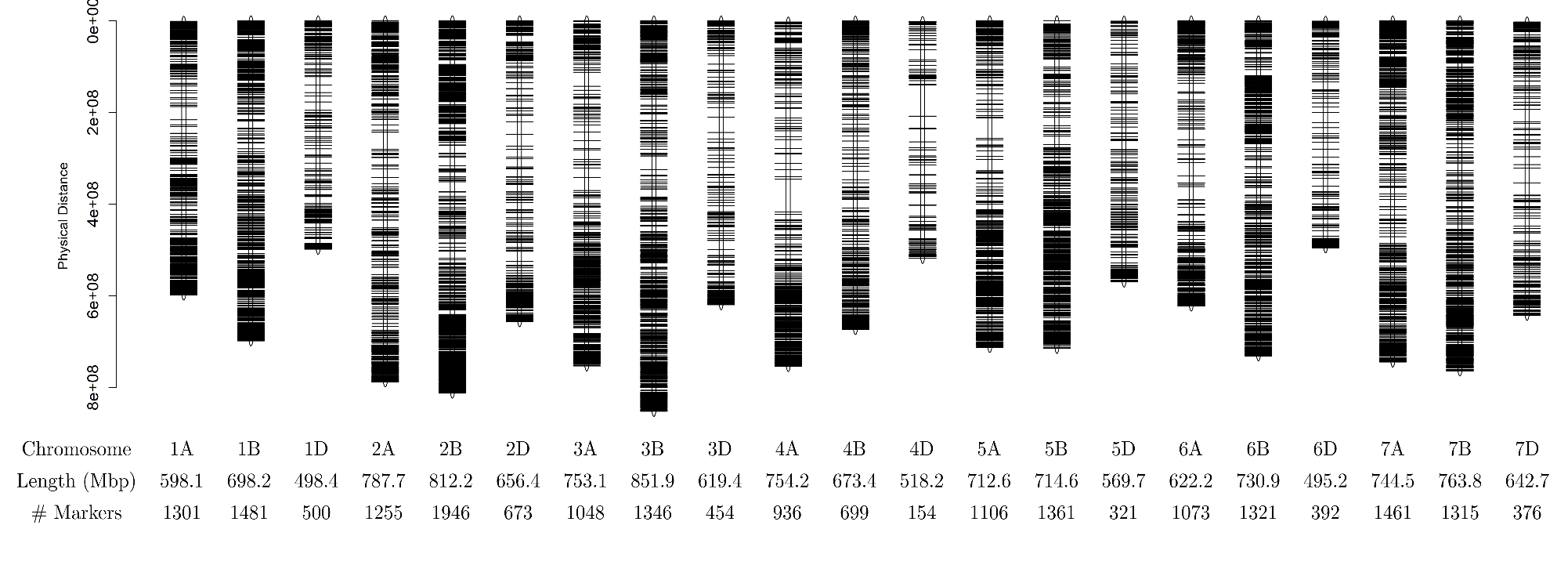


**Fig. S2** Alignment of the common markers in the physical map of the international wheat diversity panel with the consensus map derived in Wang et al. (2014). The vertical axis represents the physical map distances and horizontal axis represents the consensus map distances. Each panel represents a consensus map chromosome with common markers graphically displayed with the chromosome name from the physical map.


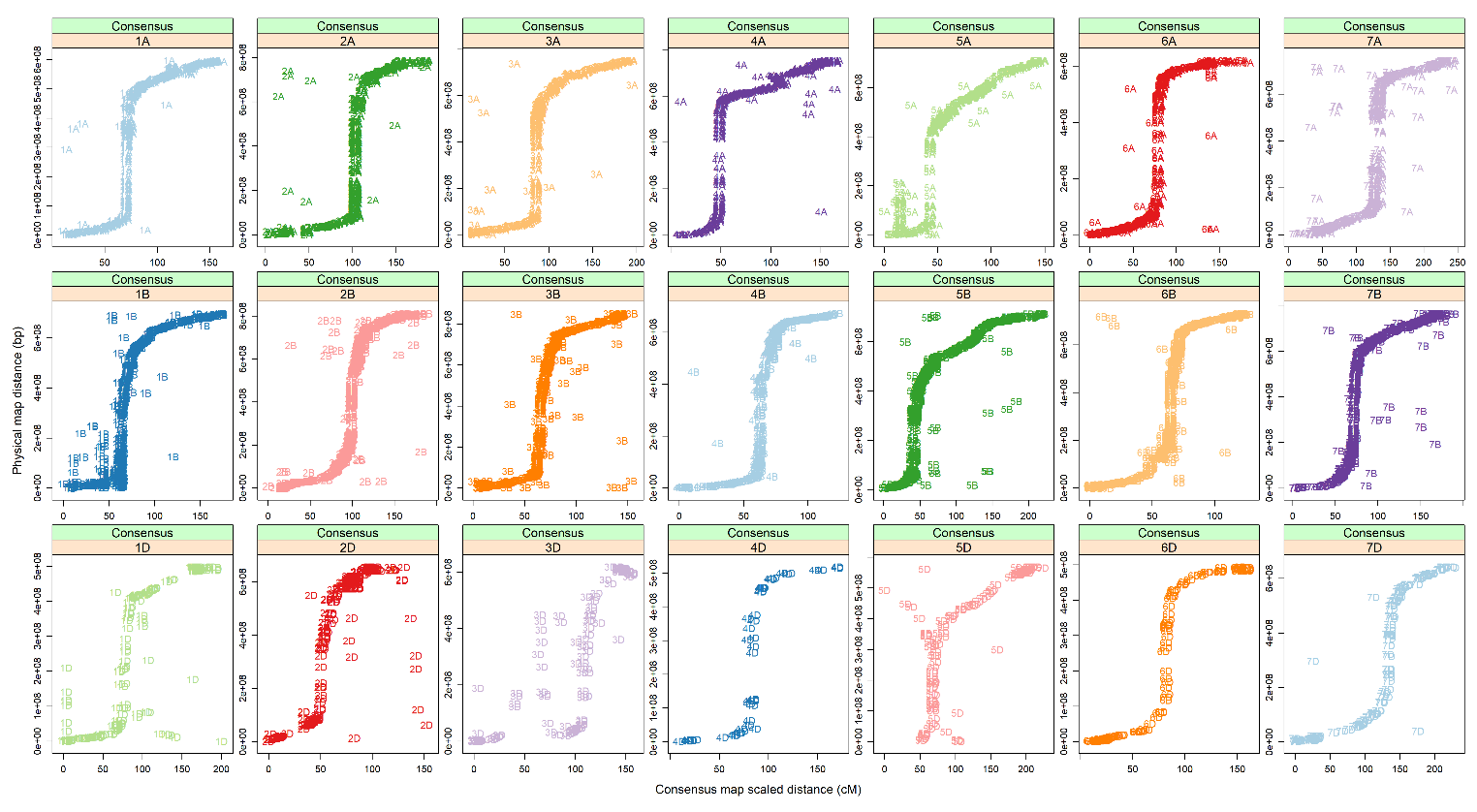


**Fig. S3** Manhattan plots of the outlier statistics extracted from the single iteration whole genome analysis of (a) pure Ptr ToxA and (b) Ptr ToxB traits scored across the international wheat diversity panel in plant bioassays. Significant marker loci linked to QTL are highlighted.


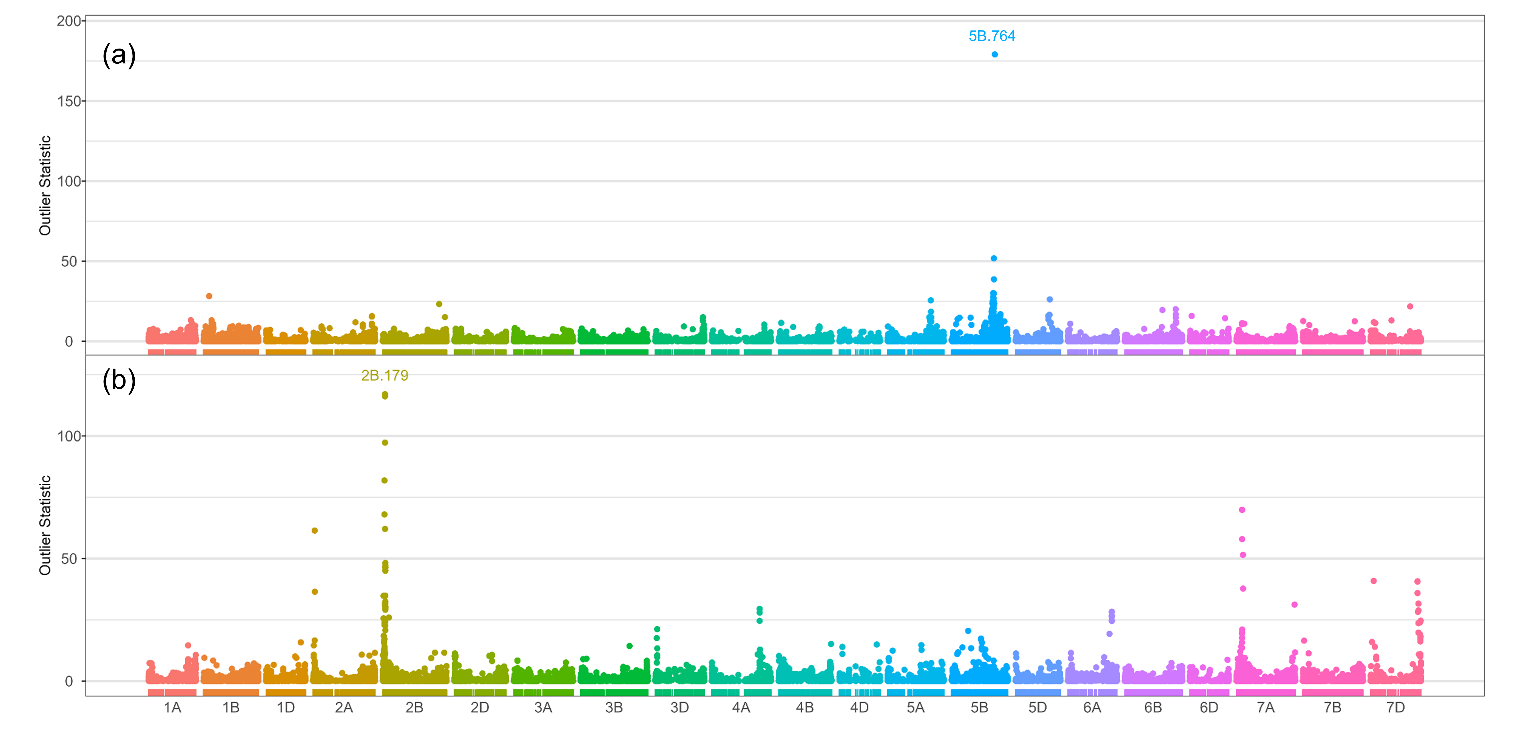


**Fig S4** Correlation plots displaying pairwise linkage disequilibrium between significant markers in Table S2. For convenience labels on the left hand side of plots contain trait names, chromosome names and markers names. Chromosomes (a) 1A-1B; (b) 2A-2D; (c) 3A-4B; (d) 5A-5D; (e) 6A-7D.


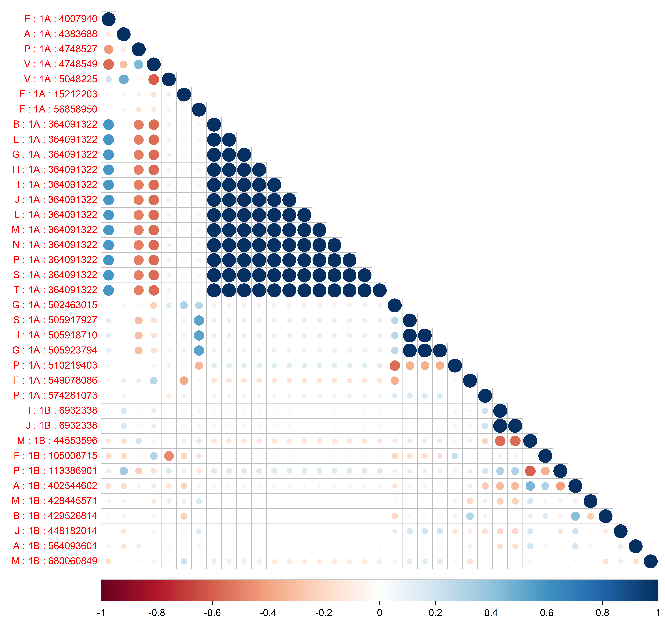

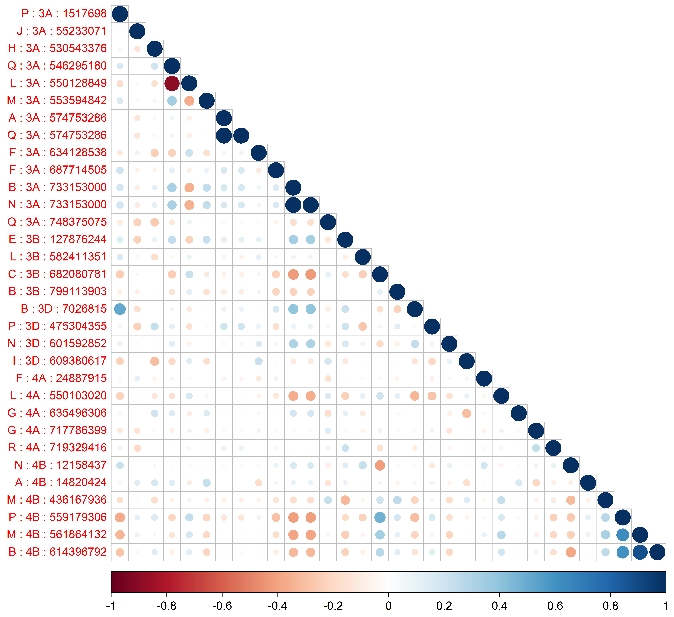

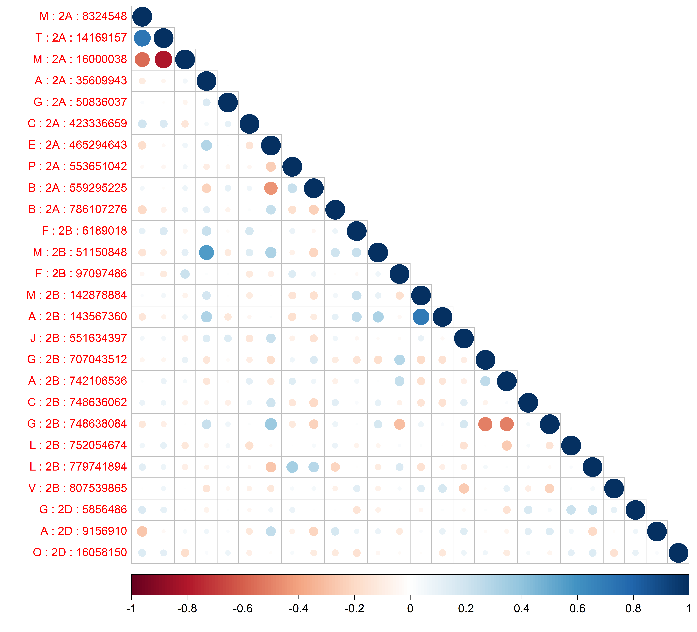

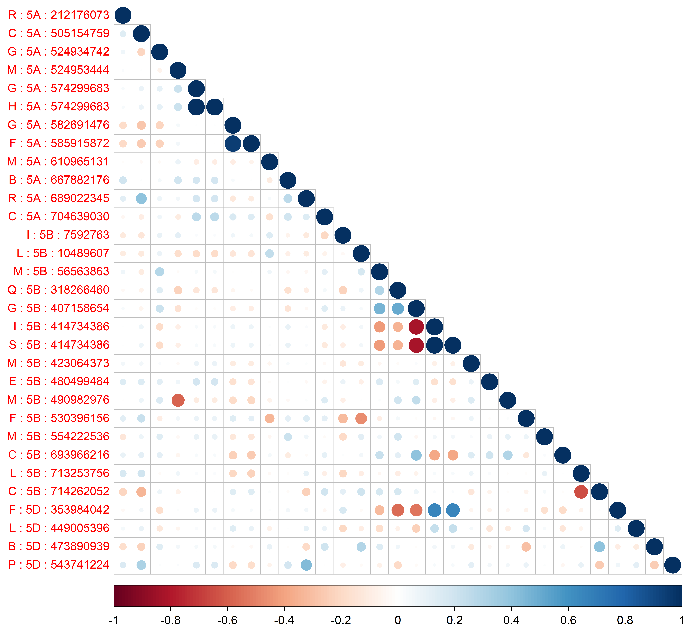

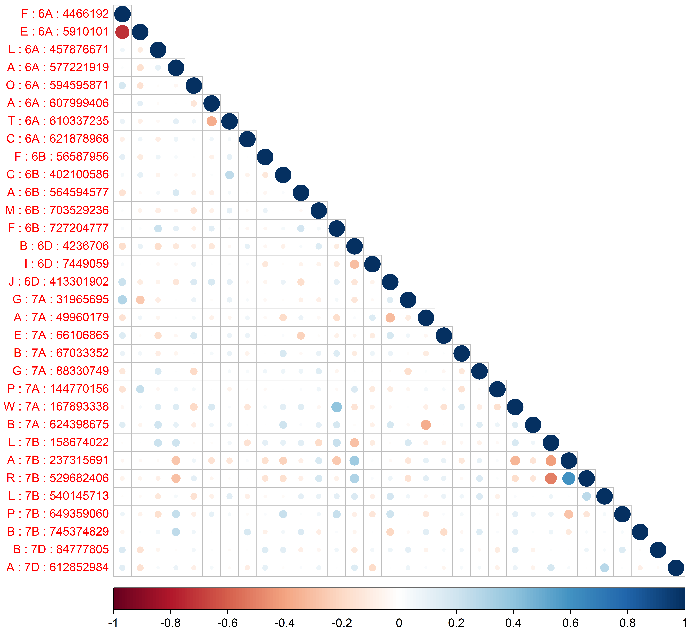


**(a)**

**(e)**

**(d)**

**(c)**

**(b)**

**Fig. S5** Overall genomic prediction accuracies for all tan spot severity traits defined in Table 2

**
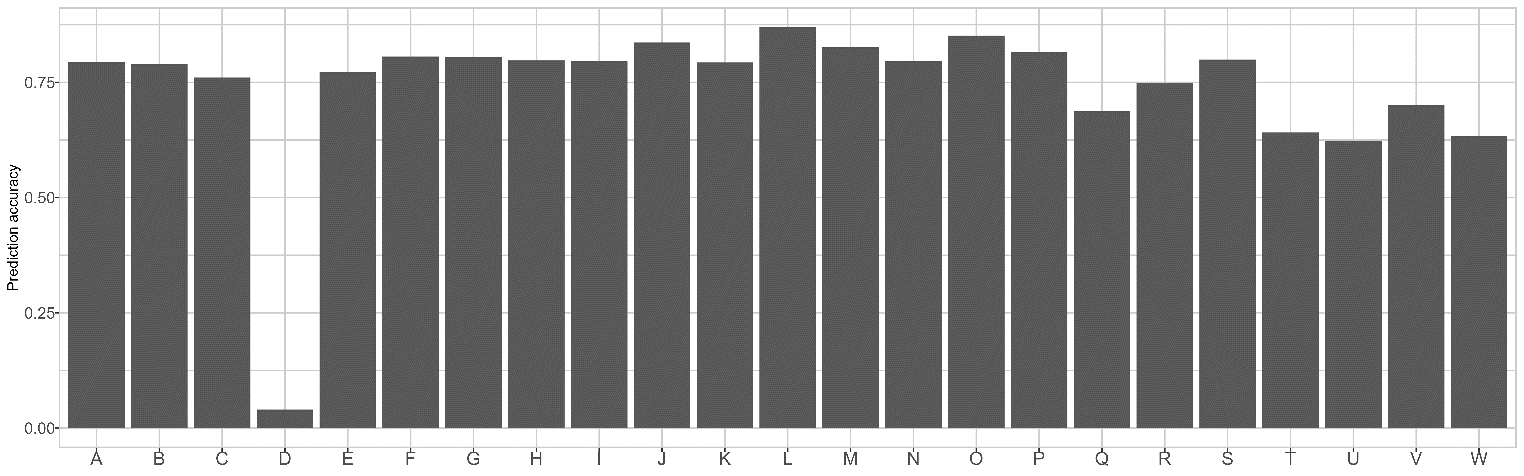
**
